# Enhancement of pullulanase production from recombinant *Bacillus subtilis* by optimization of feeding strategy and fermentation conditions

**DOI:** 10.1186/s13568-020-0948-5

**Published:** 2020-01-18

**Authors:** Yu Zhang, Yao Nie, Xia Zhou, Jiahua Bi, Yan Xu

**Affiliations:** 1 School of Biotechnology and Key Laboratory of Industrial Biotechnology of Ministry of Education, Jiangnan University, 1800 Lihu Road, Wuxi, 214122 China; 2 State Key Laboratory of Food Science and Technology, Jiangnan University, 1800 Lihu Road, Wuxi, 214122 China

**Keywords:** Pullulanase, *Bacillus subtilis*, Fermentation process, Optimization, Feeding strategy

## Abstract

Pullulanase is an important starch-debranching enzyme mostly used in starch processing-related food industries. However, the levels of pullulanase produced from recombinant *Bacillus subtilis*, a *Generally Recognized as Safe* (GRAS) host, are generally limited. To enhance the activity of pullulanase, batch fermentation and fed-batch fermentation were systematically investigated. The overall purpose is to improve the fermentation yield by optimizing the feeding strategy in the fermentation process, thereby increasing the enzyme activity of pullulanase. Therefore, in this study, the feeding methods, the feeding ingredients, the feeding concentration, and pH values were studied in detail. The optimized fermentation conditions for pullulanase production from recombinant *B. subtilis* were determined as following: inoculum volume 7%, pH 6.5, the dissolved oxygen level 30%, and constant-rate feeding of 100 mL glucose solution (400 g L^−1^) in late logarithmic growth. The OD_600_ of recombinant *B. subtilis* and enzyme activity were 84.54 and 102.75 U mL^−1^, which were respectively 141% and 144% higher than that before optimization. These findings provided a prerequisite for further amplification of the fermentation system to obtain higher enzyme activity.

## Introduction

Pullulanase (EC 3.2.1.41) is a class of debranching enzymes that catalyze the hydrolysis of alpha-1, 6-glucosidic linkages of unmodified substrates (Nakamura 1996, van der Maarel et al. 2002). The capability of the pullulanase to decompose the branches makes it widely used in various fields, which emphasizes its leading role as one of the promising new varieties of amylases (Leathers 2003). Pullulanase is widely used in the different kinds of industrial fields. In the starch industry, pullulanase and amylase can improve the efficiency of starch saccharification and reduce the cost. At present, the starch industry is based on starch raw materials, and the starch in the raw materials has nearly three-quarters of amylopectin. In addition, pullulanase also plays an outstanding role in the pharmaceutical and feed industry as well (Shiraishi et al. 1985).

*Bacillus subtilis*, as a GRAS strain, has many advantages and is used in a wide range of applications as expression host for production of food processing-related enzymes. Lack of an outer membrane and a significant bias in codon usage lead to more efficient secretion (Beaulieu et al. 2005) and allow efficient transcription and translation of the target protein. Although there are many types and sources of pullulanase that have been previously reported, there are still some limitations in using *B. subtilis* system in respect of the yield and activity of this enzyme (Kang et al. 2011, Singh et al. 2010). The approaches involving recombinant constructions have been adopted for enhancing heterologous expression of pullulanases. Deng et al. (2018) developed *B. subtilis* WB800/pMA0911-P*sacB*-*pul*-degQ (N) for pullulanase production by optimizing the enhancer of the recombinant strain, with the enzyme activity reaching 26.5 U mL^−1^ in the shake flask. Moreover, the enzyme production can be mildly enhanced by optimization of the fermentation medium and general conditions. Liu et al. optimized the fermentation medium and fermentation conditions to achieve the enzyme activity of pullulanase of 20.16 U mL^−1^ from *B. subtilis* WB600/pWB-*pul*B (Liu et al. 2012a). Since different microorganisms have their favorable process and conditions for growth and production of metabolites, it would be important to explore suitable fermentation mode and optimize fermentation process.

The level of production by biological fermentation depends not only on the performance of the production strain itself, but also on providing suitable fermentation conditions in order to fully utilize its production capacity (Kubiak et al. 2019). Optimizing the fermentation process can fully exploit the potential of the strain, improve the production efficiency of the fermentation process, and reduce the production cost (Wang et al. 2018). Therefore, the research on process optimization is especially important. In fermentation production, the reaction conditions can be artificially regulated directly or indirectly. The temperature, pH value, dissolved oxygen, and other factors in the fermentation process will affect the growth and metabolism of microorganisms. Wang et al. (2014) optimized the fermentation condition of alkaline amylase from the recombinant strain *B. subtilis* WB800, which was 3.36 times higher than that before optimization. Huang et al. (2004) have greatly improved the production of *B. subtilis* amylase by optimizing the feeding strategy.

There are many kinds of feeding strategies, which are divided into feedback feeding and non-feedback feeding. According to the feeding speed, they are divided into constant speed feeding and index feeding. By exploring different optimization fermentation methods, the production efficiency of the fermentation process can be effectively improved, leading to the lower production costs (Gao and Shi 2013). Optimal fermentation conditions and appropriate feeding strategies are critical for cell growth and protein expression (Yadav et al. 2017).

In this work, we studied the differences between batch fermentation and fed-batch fermentation, and further explored the characteristics of two feeding strategies, DO-stat feeding strategy and constant-rate feeding strategy. The optimum feeding carbon source and optimum concentration were determined by adding different concentrations of glucose and sucrose under constant-rate feeding. Afterwards, we investigated the effects of fermentation system pH value on the process. Finally, the enzyme activity of the recombinant pullulanase was determined after combining the optimized fermentation conditions.

## Materials and methods

### Strains, media, and growth conditions

*B. subtilis* WB800-P_*HpaII*_-*pul* used in this study was constructed in our laboratory (Wang et al. 2019a, b). *B. subtilis* WB800 was employed for the host for recombinant pullulanase production. Pullulanase (GenBank Accession No. JN872757) was from *B. naganoensis*. The pullulan polysaccharide used to determine the activity of pullulanase was purchased from Tokyo Kasei Kogyo Co., Ltd (Tokyo, Japan). Prime STAR Max DNA Polymerase was obtained from TaKaRa Biotechnology Co., Ltd. (Dalian, China). The DNA primers were obtained from Shanghai Sangon Biological Engineering Technology & Services Co., Ltd. (Shanghai, China). All other reagents were of analytical grade and were commercially available unless otherwise indicated.

### Media and feeding solutions

Luria-Bertani (LB) broth or agar plate was composed for 10 g L^−1^ tryptone, 5 g L^−1^ yeast extract, and 10 g L^−1^ NaCl, supplementing with 2% agar power. Seed cultures were grown in medium that contained sucrose 40 g L^−1^, soy peptone 30 g L^−1^, KH_2_PO_4_ 6 g L^−1^, MgCl_2_·6H_2_O 2.04 g L^−1^. The composition of fermentation medium were sucrose 70 g L^−1^, soy peptone 50 g L^−1^, KH_2_PO_4_ 5 g L^−1^, and MgCl_2_·6H_2_O 3.06 g L^−1^. The feeding solutions of fed-bath fermentation contained different concentrations of glucose solutions (300 g L^−1^, 400 g L^−1^, and 500 g L^−1^) and sucrose solutions (400 g L^−1^).

### Seed culture

Frozen glycerol strain stored at − 20 °C was streaked into LB solid plates, and then activated by incubating at 37 °C for 10–12 h. A single colony was picked into test tube, which contained 5 mL seed culture medium, culturing at 37 °C, 220 r min^−1^ on a constant temperature shaker for 10–12 h The activated seed solution was transferred into 500 mL shake flasks containing 50 mL of seed culture medium on a rotary shaker. The inoculum amount was 7% (v/v), and cultured at 37 °C, 220 r min^−1^ on a constant temperature shaker for 8–10 h.

### Bioreactor fermentation

Bioreactor cultivation was performed in a 3 L fermenter (BioFlo110, New Brunswick Scientific co., Inc.). The liquid volume was 1 L, the ventilation volume was 1.5 vvm, and the initial stirring speed was 300 rpm. The 7% seed culture was added to the modified semisynthetic medium for batch or fed-batch cultivation. The fed-batch fermentation required feeding carbon source. According to different methods, we chose the DO-Stat feeding strategy and the constant-rate feeding strategy, feeding 100 mL sucrose solution (400 g L^−1^) separately. The biomass and enzyme activity were monitored regularly. Considering the effect of the carbon source type on the fermentation, we selected glucose solution (400 g L^−1^) as feeding solution for comparison with sucrose solution (400 g L^−1^). Then, in order to determine the optimal carbon source concentration, we set up three concentration gradients of 300 g L^−1^, 400 g L^−1^, and 500 g L^−1^ for experiments, respectively. According to the relevant report, the addition of carbon source can affect the acidity and alkalinity of the fermentation broth (Wang et al. 2018), and thus the pH was finally optimized.

### Biomass of *B. subtilis* and enzyme activity assay

Biomass was monitored by measuring the optical density at 600 nm (OD_600_). After appropriately diluting the fermentation broth containing the cells, the values of OD_600_ were measured using a 721 UV–Vis spectrophotometer (Labbeiki et al. 2014).

Enzymatic activity of pullulanase was determined by measuring the generated aldehyde groups released from enzymatic saccharification with pullulan as the substrate (Chen et al. 2012). We sampled regularly during the fermentation process and centrifuged for 10 min at 12,000 rpm, then collected the supernatant and diluted to a suitable multiple with buffer solution (0.1 M, pH 4.5 sodium acetate and acetic acid buffer). 200 μL of 2% pullulan substrate and 200 μL of the diluted fermentation broth were mixed and placed in a water bath at 60 °C for 20 min as an experimental group. The control group added 200 μL of substrate after the reaction. The two groups were added with 600 μL of 3,5-dinitrosalicylic acid (DNS), placed in a boiling water bath for 5 min, cooling down and then added with 3 mL of ultrapure water. 200 μL of the sample was transferred in 96-well plate and absorbance at 540 nm wavelength was measured in microplate reader. Three parallel experiments were set up in each group and the results were finally averaged. One unit of pullulanase enzyme activity was defined as the amount of enzyme required to catalyze the decomposition of the substrate of pullulan to produce a reducing sugar (equivalent to 1 μmol of glucose) per minute under the reaction conditions.

## Results

### Cultivation of recombinant strain by batch fermentation

**Fig. 1:**
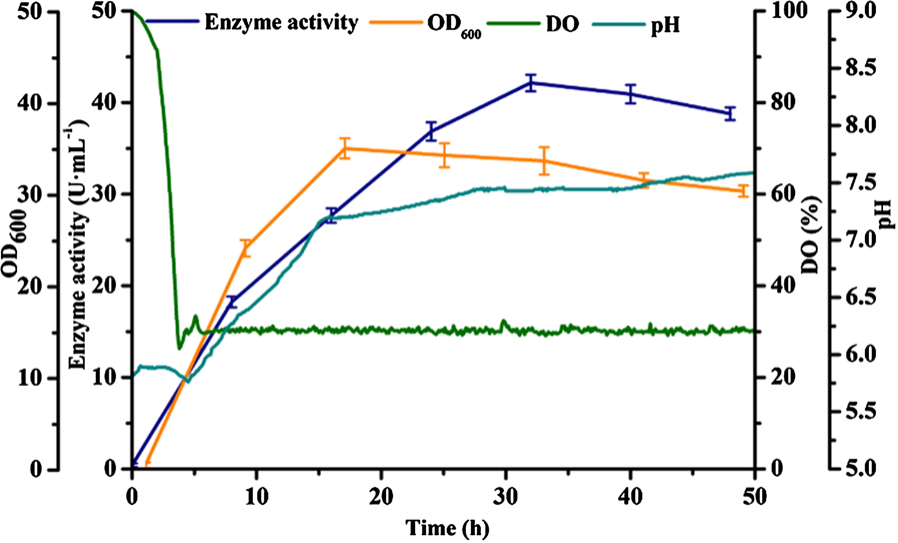
Batch fermentation of the recombinant strain *B. subtilis* WB800-P_*HpaII*_-*pul*. The values of OD_600_ (orange curve), extracellular enzyme activity (blue curve), dissolved oxygen (green curve), and pH (light blue curve) were measured during the fermentation process. The averages of three independent experiments together with the corresponding standard deviations are shown for all values of OD_600_ and enzyme activity

The strain *B. subtilis* WB800-P_*HpaII*_-*pul* was cultured in 3 L fermenter for 50 h. During the batch fermentation process, the temperature and dissolved oxygen level were maintained at 37 °C and 30%, respectively, and pH value was natural. The biomass and enzyme activity were measured at regular intervals. As shown in Fig. 1, the recombinant strain showed slow growth in the first 2 h, and then it grew rapidly from the next 14 h. The biomass did not increase after the cells entered the stable phase; the maximum value of OD_600_ was 35.12. After fermentation for 32 h, the enzyme activity reached the maximum value of 42.15 U mL^−1^. In the early stage of fermentation, the enzyme activity of pullulanase increased remarkably with increasing the recombinant strain concentration. After 32 h, the enzyme activity decreased slightly but overall remained unchanged.

In general, heterologous protein expression lagged behind the growth of *B. subtilis* and was closely related to it. During the fermentation process, the pH value continued to rise with the consumption of the carbon source, reaching about 7.5 at 50 h. The consumption of nutrients and the accumulation of metabolic waste in the late stage of fermentation might be the main reasons for limiting the growth of the recombinant strain and expression of heterologous protein (Liu et al. 2008). With the purpose of providing sufficient carbon sources, we intended to optimize the feeding way and fed-batch strategy of fermentation process in the next experiment.

### Effects of feeding ways of fed-batch fermentation

In order to obtain high-density recombinant cell concentrations during fermentation process and avoid substrate inhibition and accumulation of metabolic product, fed-batch fermentation strategy was proposed. By adding fresh feeding to the bioreactor, it would be feasible to overcome the premature termination of fermentation due to insufficient nutrients (Park et al. 1992). Fed-batch culture was a method that referred to the intermittent or continuous addition of one or more specific restricted substrates to the fermenter during batch culture until the end of the fermentation process followed by the discharge of culture (Son et al. 2007).

To study the effect of feeding ways on the production of pullulanase, we have adopted two common strategies: constant-rate feeding (non-feedback feeding strategy) and DO-stat feeding (feedback feeding strategy) (Table 1). The constant-rate feeding strategy was easy to operate, and the feeding was continuously fed at a constant rate (Prentice et al. 2007). The source continuously supplied energy to the cells, which could meet the needs of growth and expression of foreign proteins. The second feeding strategy was more intuitive to reflect the state of fermentation (Zhu et al. 2015). It was monitored by the on-line detection device of dissolved oxygen, and once the dissolved oxygen rebounded, the feeding was added to maintain the stability of DO. Both methods had their own advantages; therefore, we performed further experiments to find out which fermentation process was more suitable for this study.

**Table 1:** Comparison of two feeding strategies

Strategy	Time	Rate (mL min^−1^)	Volume (mL)	Ingredient
DO-stat feeding	DO rebound period	0.1	100	Sucrose
Constant-rate feeding	Late logarithmic growth	0.1	100	Sucrose

**Fig. 2:**
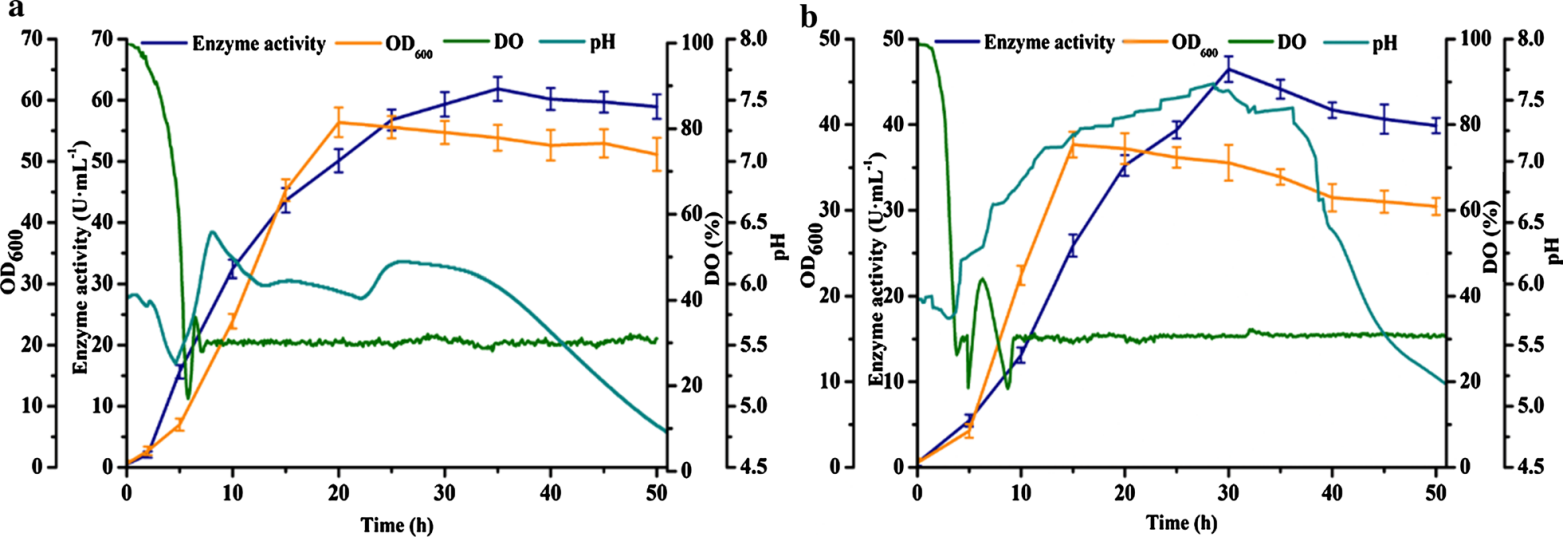
Fed-batch fermentation of the recombinant strain *B. subtilis* WB800-P_*HpaII*_-*pul* by DO-Stat feeding (**a**) and constant-rate feeding (**b**). The values of OD_600_ (orange curve), enzyme activity (blue curve), dissolved oxygen (green curve), and pH (light blue curve) were measured during the fermentation process. The averages of three independent experiments together with the corresponding standard deviations are shown for all values of OD_600_ and enzyme activity

After carbon in the fermentation broth was consumed in a large amount, the oxygen consumption of the cells decreased due to the lack of nutrients, and the dissolved oxygen concentration rose rapidly (Liu et al. 2012b). However, the dissolved oxygen concentration decreased again after the addition of carbon. In this way, the dissolved oxygen concentration could be maintained at a constant level. For DO-Stat feeding fed-batch fermentation, the dissolved oxygen gradually decreased during the initial stage of fermentation. As shown in Fig. 2a, when the dissolved oxygen was reduced to 30%, it was coupled with the rotational speed, and soon after, the dissolved oxygen was restored to 30%. When the dissolved oxygen suddenly rose, the carbon source was added. When fermenting for 15 h, the maximum value of OD_600_ was 37.68 and when fermenting for 30 h, the enzyme activity reached the maximum value of 46.49 U mL^−1^.

For constant-rate feeding, the biomass of *B. subtilis* increased dramatically in the logarithmic growth phase, so that the carbon source was quickly consumed. Feeding time earlier or later was not conducive to the accumulation of the target product. Feeding at an earlier time would stimulate cell growth and accelerate consumption of carbon source; on the contrary, feeding at a deferred time would lead to insufficiency of nutrients and postpone the growth of the recombinant strain. By supplementing more carbon source, the OD_600_ and enzyme activity were also increased. As shown in Fig. 2b, similar to the batch fermentation process, the recombinant strain transferred to the fermenter needed to be adapted for a period. The cell concentration of the recombinant strain increased dramatically, the maximum value of OD_600_ was 56.4 when fermenting for 20 h and the enzyme activity reached the maximum value of 61.86 U mL^−1^ when fermenting for 35 h. Compared with batch fermentation, the concentration of the recombinant strain and enzyme activity of fed-batch fermentation both increased. In terms of enzyme activity, the two feeding fermentations were increased by 10.3% and 46.8%, respectively. This result indicated that carbon source deficiency was a major factor limiting the fermentation process.

### Effect of feeding carbon source

**Fig. 3:**
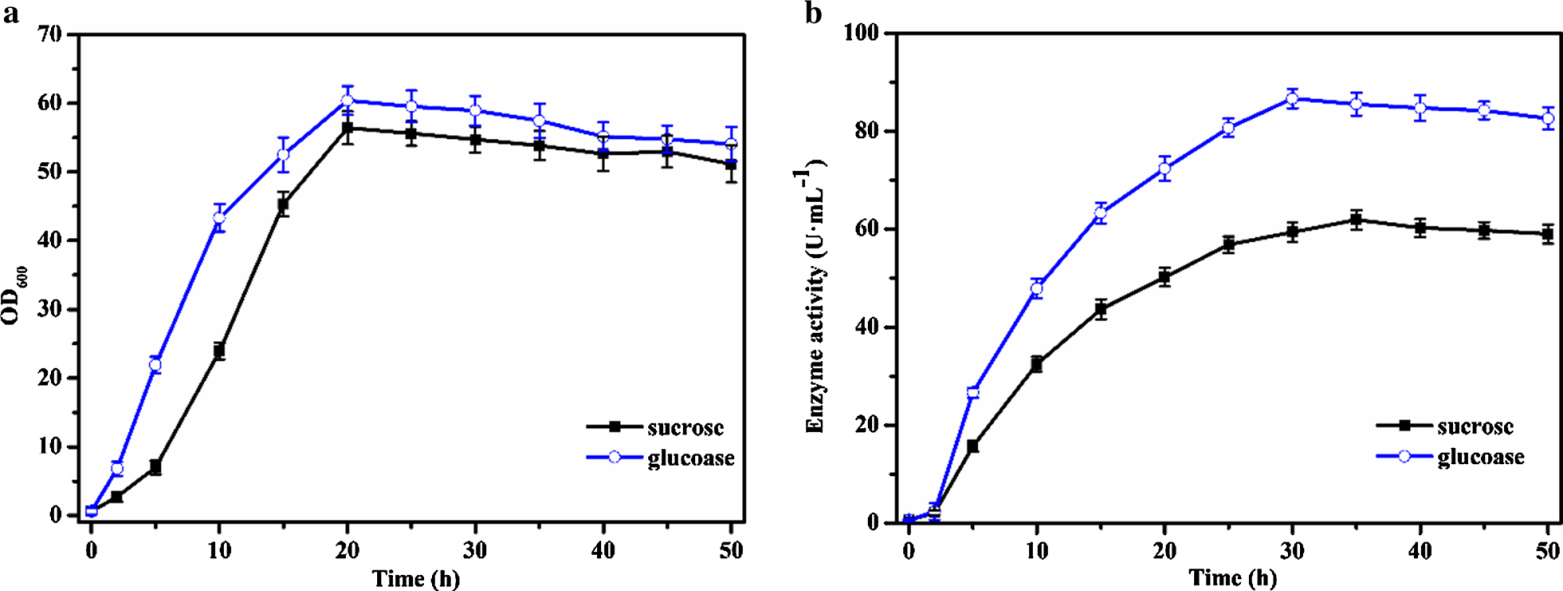
Effect of feeding carbon source on OD_600_ (**a**) and enzyme activity (**b**). Glucose solution (400 g L^−1^) (blue curve) and sucrose solution (400 g L^−1^) (black curve) were used as the feeding carbon sources. The averages of three independent experiments together with the corresponding standard deviations are shown for all values of OD_600_ and enzyme activity

To explore the effect of the feeding ingredients on the fermentation, we chose sucrose (the initial carbon source used in fermentation medium) and glucose (the most commonly used carbon source) as the carbon sources for fed-batch fermentation of the recombinant strain *B. subtilis* WB800-P_*HpaII*_-*pul*. As shown in the Fig. 3a, the maximum values of OD_600_ were 60.4 and 56.4 when feeding glucose and sucrose, respectively; while in the Fig. 3b, the enzyme activities reached the maximum values of 86.65 U mL^−1^ and 61.86 U mL^−1^ when feeding glucose and sucrose, respectively. The addition of carbon source was conducive to the growth of *B. subtilis*, indicating that the initial carbon source in the medium may not be sufficient for the fermentation process. Obviously, glucose was significantly better than sucrose for cell growth and production of enzyme from recombinant strain. As known, glucose is more easily used than sucrose, and thus improved the fermentation significantly. When glucose was fed, the extracellular enzyme activity reached 86.65 U mL^−1^ during the fermentation for 30 h, which was 105.6% higher than the batch fermentation. Therefore, glucose was added to achieve the purpose of timely supplementing carbon source.

### Effect of feeding concentration

Feeding concentration was one of the key parameters in the fermentation of pullulanase, which influenced the cell growth and distribution of metabolites (Xiao et al. 2017). We optimized the feeding concentration based on the determination of the optimum feeding rate, selecting different gradients of 300 g L^−1^, 400 g L^−1^, and 500 g L^−1^, respectively.

**Fig. 4:**
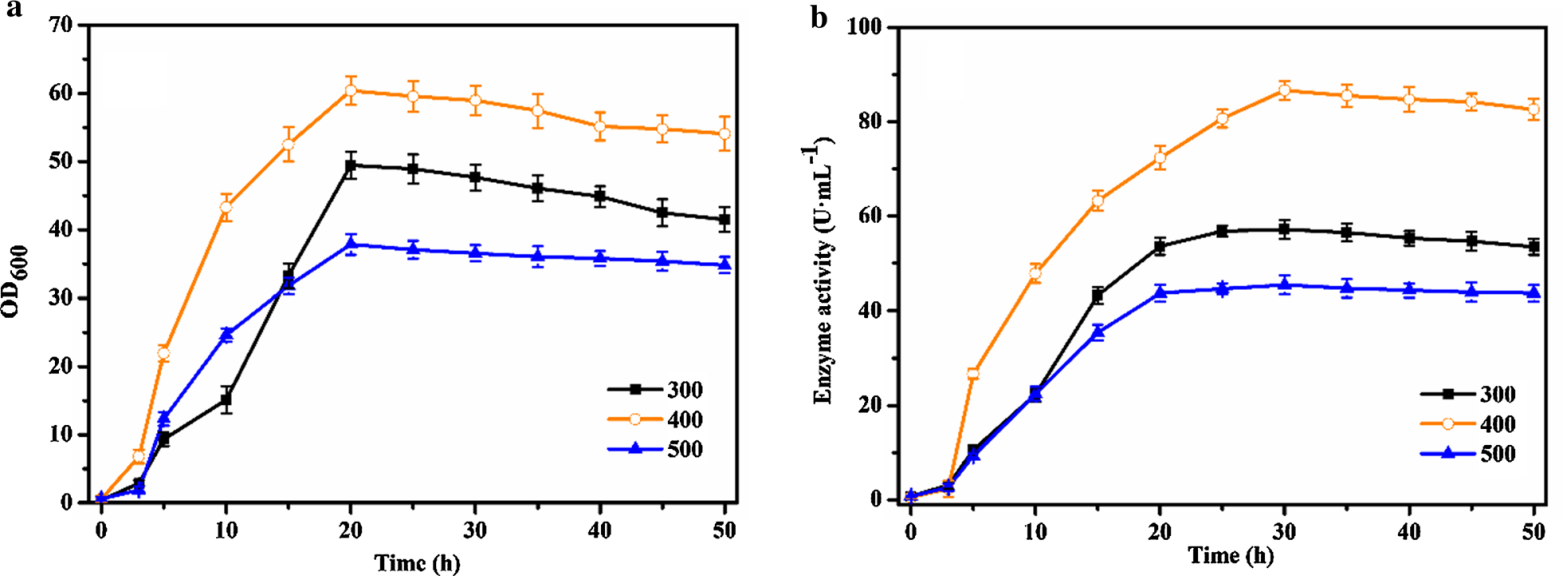
Effect of feeding concentration on OD_600_ (**a**) and enzyme activity (**b**). Glucose was fed at 300 g L^−1^ (black curve), 400 g L^−1^ (orange curve), and 500 g L^−1^ (blue curve), respectively. The averages of three independent experiments together with the corresponding standard deviations are shown for all values of OD_600_ and enzyme activity

**Fig. 5:**
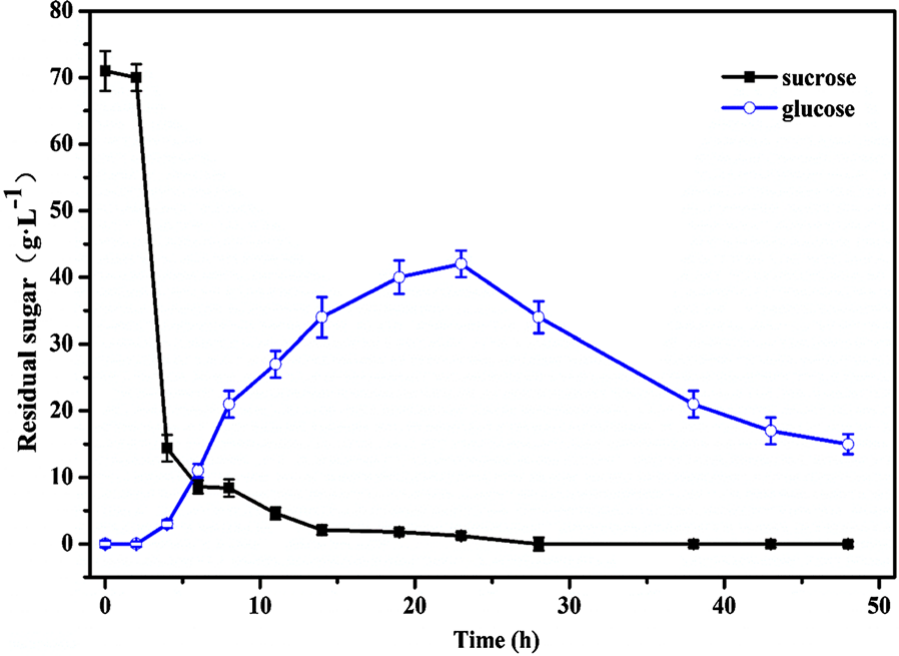
Residual sugar including glucose (blue curve) and sucrose (black curve) during fed-batch fermentation by feeding 500 g L^−1^ glucose solution. The averages of three independent experiments together with the corresponding standard deviations are shown for all values of OD_600_ and enzyme

As shown in the Fig. 4a, the maximum values of OD_600_ were 49.48, 60.4, and 37.85 when feeding glucose at 300 g L^−1^, 400 g L^−1^, and 500 g L^−1^, respectively; while in the Fig. 4b, the corresponding enzyme activities reached the maximum values of 57.2 U mL^−1^, 86.65 U mL^−1^, and 45.42 U mL^−1^ when fermenting for 30 h, respectively. The profiles of cell growth and enzyme production exhibited similar trends. When the feeding concentration was 400 g L^−1^, the biomass and the enzyme activity both reached the highest values. When the feeding concentration was lower, it was not favorable for cell growth and enzyme production. However, when glucose solution (500 g L^−1^) was added at 0.1 mL min^−1^ at logarithmic growth phase (8 h), combining with the glucose generated from sucrose hydrolysis at the beginning stage of fermentation, glucose was further accumulated to the maximum concentration of 42 g L^−1^ during fermentation from 8 to 25 h, then was gradually consumed during the late logarithmic growth phase, resulting in decrease of glucose concentration to a detectable level about 20 g L^−1^ even in the stationary phase. During the fed-batch fermentation process, therefore, the glucose concentration increased at first and then decreased after fermentation for 25 h (Fig. 5). Because the residual sugar was continuously maintained at a detectable level even higher than 20 g L^−1^ in the stationary phase, which might have an inhibitory effect on cell growth and production of target enzyme, feeding glucose at 500 g L^−1^ was not favorable for the improvement of both biomass and enzyme activity. Based on the results of feeding concentration optimization, feeding glucose at 400 g L^−1^ was adopted for fed-batch fermentation of the recombinant strain *B. subtilis* WB800-P_*HpaII*_-*pul*.

### Effect of pH on fermentation

In batch culture, the pullulanase activity was 42.15 U mL^−1^ with the OD_600_ value of about 35, where the pH at the end of fermentation was about 7.5 (Fig. 1); When feeding with sucrose and glucose, the OD_600_ value was increased to about 60, while the pH at the end of the fermentation was 5 or less (Fig. 2). Because the pH value changed obviously during the fermentation process for both batch fermentation and fed-batch fermentation, it would be necessary to investigate the effect of constant pH on cell growth and enzyme production by controlling the pH of the fermentation broth.

**Fig. 6:**
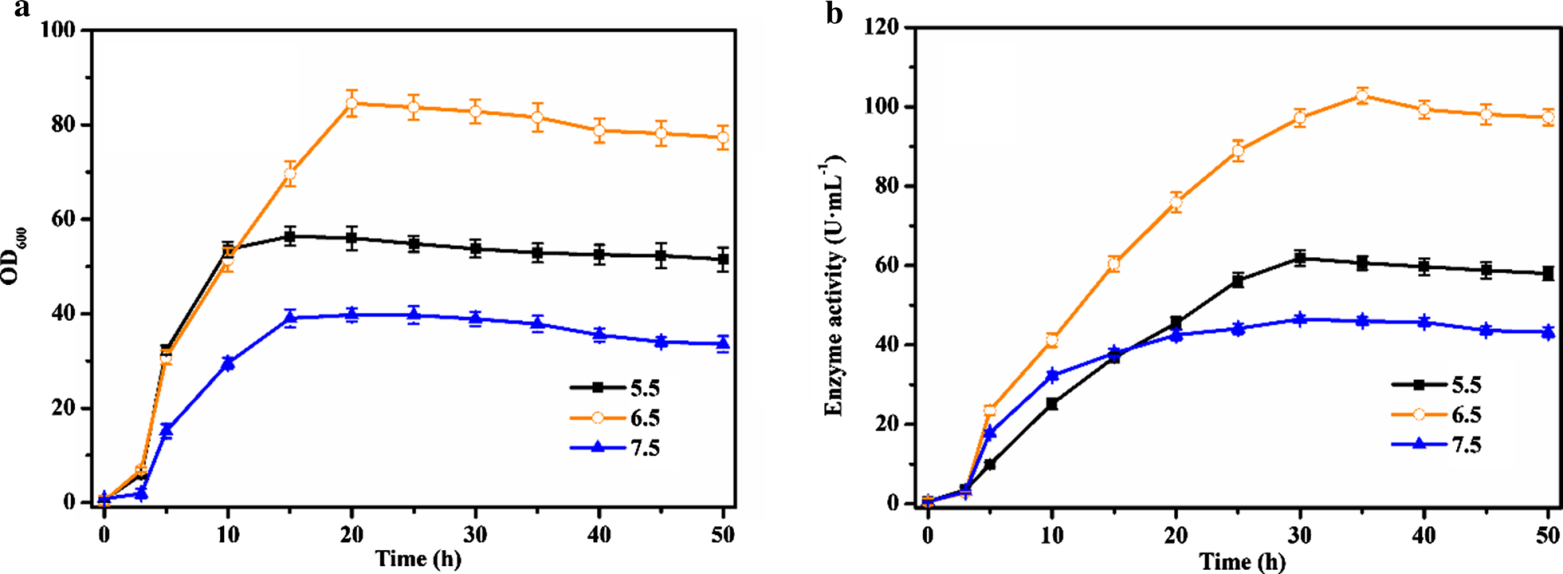
Effect of controlled pH on OD_600_ (**a**) and enzyme activity (**b**). The pH value of fermentation broth of *B. subtilis* WB800-P_*HpaII*_-*pul* was adjusted constantly at pH 5.5 (black curve), pH 6.5 (orange curve), and pH 7.5 (blue curve), respectively. The averages of three independent experiments together with the corresponding standard deviations are shown for all values of OD_600_ and enzyme activity

As shown in Fig. 6, the overall trend of cell growth and enzyme production was consistent. The cell density reached a maximum at about 15–20 h of fermentation, and the enzyme activity reached a maximum at about 30 h of fermentation. However, the growth rate of the cells and the rate of the pullulanase synthesis were different, which might be related to pH values. When the pH value was 5.5, the cells grew by a sharp speed within 3–15 h to reach a stable phase. At 20 h, the maximum OD_600_ was 39.76 and the concentration of the cells remained unchanged. The enzyme activity reached a maximum of 46.5 U mL^−1^ during 30 h of fermentation. When the pH value was 6.5, the recombinant strain continued to grew at a faster speed. The OD_600_ reached a maximum of 84.54 at 20 h of fermentation, and the maximum enzyme activity reached 102.75 U mL^−1^ after 35 h of fermentation. When the pH value was 7.5, the OD_600_ reached a maximum of 39.76 after 20 h of fermentation, and the maximum enzyme activity reached 46.5 U mL^−1^ after 30 h of fermentation. The pH value was an important parameter to ensure the normal reproduction and metabolism of microorganisms, affecting the fermentation significantly.

## Discussion

In this study, batch fermentation and feeding fermentation were firstly compared. The recombinant strain concentration and enzyme activity of the feeding fermentations were better than the batch fermentation. The enzyme activity of DO-stat feeding strategy increased by 10.3% and the enzyme activity of constant-rate feeding strategy increased by 46.8%, compared with batch fermentation.

Concerning the feeding mode, we used two methods for comparison, DO-Stat feeding and constant-rate feeding (Ding et al. 2014). Both feeding strategies were conducive to the accumulation of fermentation products. After comparison, it was found that constant-rate feeding in the logarithmic growth was better for growth. When the carbon in the medium was exhausted, the rate of aerobic metabolism of the recombinant strain was slowed down, resulting in a rapid increase in dissolved oxygen in the medium, and the dissolved oxygen rapidly decreased after the addition of carbon. Due to this observation, the agitation rate was controlled by dissolved oxygen feedback to maintain the proper level of nutrients in the medium. However, because this method had certain retardation and could not contemporary respond to the state in the fermenter, the feeding was slightly later than the ideal feeding time. In contrast, constant-rate feeding began to feed in late logarithmic growth, making up for the shortcomings of the former method and more suitable for the fermentation process.

Based on the optimization of feeding mode, it was necessary to explore the feeding ingredients and feeding concentrations for the further improvement of biomass and enzyme activity. By optimizing the feeding components, glucose was better than sucrose for accumulation of biomass and enzyme activity from the recombinant strain, with the increased OD_600_ of 60.4 and the enzyme activity of 86.65 U mL^−1^. Therefore, glucose can be added to achieve the purpose of timely supplementing carbon source. In addition, the phenomenon that microbes adopt different nutrient intake methods can be explained by the rational application of metabolic network topology and protein resources (Wang et al. 2019a, b). By investigating the effect of feeding concentration of glucose, when the glucose solution was 400 g L^−1^, it was beneficial to biomass and enzyme activity of the recombinant strain.

The addition of glucose solution had a great influence on the pH value in the fermentation process, making the recombinant strain more susceptible to acidic substances. The pH value affected the enzyme activity in the microbial cells and the charge of the microbial cell plasma membrane, as well as the dissociation of certain important nutrients and intermediate metabolites in the medium (Duan et al. 2006). When the pH was 6.5, the OD_600_ reached a maximum of 84.54 at 20 h of fermentation, and the maximum enzyme activity reached 102.75 U mL^−1^ after 35 h of fermentation, giving the OD_600_ and the enzyme activity of 39.9% and 18.6% increases compared to the fermentation under uncontrolled pH, respectively.

Consequently, by optimizing fermentation conditions including feeding mode, feeding amount, feeding composition, and pH value, the optimal conditions for pullulanase production from recombinant *B. subtilis* were obtained: inoculum volume 7%, pH value 6.5, dissolved oxygen level 30%, constant-rate feeding of 400 mL glucose solution (400 g L^−1^) in late logarithmic growth. Finally, the production of the pullulanase was efficiently enhanced and the enzyme activity of 102.75 U mL^−1^ was obtained with OD_600_ of 84.54. Therefore, the enzyme activity was greatly improved by optimizing the feeding strategy and the corresponding fermentation conditions (Deng et al. 2018, Liu et al. 2012a, b), which provides a prerequisite for further amplification of the fermentation system to obtain higher enzyme activity.

## Data Availability

The data supporting the conclusions of this article are included within the article. Data and materials can also be requested from the corresponding author.

## Abbreviations

GRAS: *Generally Recognized as Safe*
DO: dissolved oxygen
OD_600_: optical density at 600 nm
LB: Luria-Bertani broth or agar plates
DNS: 3, 5-dinitrosalicylic acid
